# Macrophage Dysregulation and Impaired Skin Wound Healing in Diabetes

**DOI:** 10.3389/fcell.2020.00528

**Published:** 2020-06-26

**Authors:** Pijus K. Barman, Timothy J. Koh

**Affiliations:** Department of Kinesiology and Nutrition, Center for Wound Healing and Tissue Regeneration, University of Illinois at Chicago, Chicago, IL, United States

**Keywords:** monocytes, macrophages, diabetes, wound healing, bone marrow, inflammation, hematopoietic stem, progenitor cells

## Abstract

Monocytes (Mo) and macrophages (Mϕ) play important roles in normal skin wound healing, and dysregulation of wound Mo/Mϕ leads to impaired wound healing in diabetes. Although skin wound Mϕ originate both from tissue resident Mϕ and infiltrating bone marrow-derived Mo, the latter play dominant roles during the inflammatory phase of wound repair. Increased production of bone marrow Mo caused by alterations of hematopoietic stem and progenitor cell (HSPC) niche and epigenetic modifications of HSPCs likely contributes to the enhanced number of wound Mϕ in diabetes. In addition, an impaired transition of diabetic wound Mϕ from “pro-inflammatory” to “pro-healing” phenotypes driven by the local wound environment as well as intrinsic changes in bone marrow Mo is also thought to be partly responsible for impaired diabetic wound healing. The current brief review describes the origin, heterogeneity and function of wound Mϕ during normal skin wound healing followed by discussion of how dysregulated wound Mϕ numbers and phenotype are associated with impaired diabetic wound healing. The review also highlights the possible links between altered bone marrow myelopoiesis and increased Mo production as well as extrinsic and intrinsic factors that drive wound macrophage dysregulation leading to impaired wound healing in diabetes.

## Introduction

Skin wound healing involves distinct but overlapping phases of hemostasis, inflammation, proliferation, and remodeling (Eming et al., 2014). Immediately following injury, platelet aggregation is important to achieve coagulation and hemostasis in the wound. The inflammatory phase is induced by pro-inflammatory mediators released by injured tissues, and is critical for controlling infection, clearing necrotic debris, and induction of the wound healing process (Zhang and Mosser, 2008; Koh and DiPietro, 2011; Eming et al., 2017). Next, the proliferative or tissue formation phase involves proliferation of a number of cell types to form a provisional connective tissue matrix, new blood vessels, and epithelial closure. Finally, during the remodeling phase the newly formed tissues are remodeled to improve their integrity (Eming et al., 2014). A diverse set of cells such as platelets, mast cells, neutrophils, monocytes (Mo), macrophages (Mϕ), lymphocytes, keratinocytes, fibroblasts, and endothelial cells all contribute to the process of skin wound healing (Canedo-Dorantes and Canedo-Ayala, 2019).

Among all cell types Mo/Mϕ play critical roles in each phase of wound repair through host defense, tissue debridement and cell regulatory functions (Goren et al., 2009; Mirza et al., 2009; Lucas et al., 2010; Boniakowski et al., 2017). Studies using *LysM-Cre/DTR* genetically modified mice that allow for inducible depletion of Mo/Mϕ by diphtheria toxin (DT) administration provide strong evidence that these cells are required for normal wound healing, promoting angiogenesis, collagen deposition, and closure (Goren et al., 2009; Mirza et al., 2009; Lucas et al., 2010).

Properly regulated numbers and phenotypes of Mo/Mϕ are crucial for efficient wound repair, and the dysregulation of either may lead to impaired wound healing. For example, increased numbers of wound Mo/Mϕ have been shown to be associated with impaired wound healing in diabetes (Mirza and Koh, 2011; Bannon et al., 2013; Barman et al., 2019b). Similarly, an impaired transition from pro-inflammatory into pro-healing wound Mo/Mϕ phenotypes and reduced phagocytic ability contribute to chronic inflammation and impaired wound healing in diabetes (Mirza and Koh, 2011; Bannon et al., 2013; Mirza et al., 2013, 2014; Gallagher et al., 2015; Yan et al., 2018; Barman et al., 2019b). This brief review considers the origin, heterogeneity and function of wound Mϕ during normal wound healing followed by discussion of how dysregulation of numbers and phenotypes of wound Mϕ may lead to impaired diabetic wound healing. The review also highlights the possible links between altered bone marrow myelopoiesis, wound macrophage dysfunction and impaired wound healing, and finally highlights gaps in the current literature, whose filling could lead to new therapeutic interventions for diabetic wounds.

## Origin of Skin Wound Mϕ

Skin wound Mϕ originate both from tissue resident Mϕ and infiltrating Mo with significantly larger contribution from the latter (Davies et al., 2013; Malissen et al., 2014; Minutti et al., 2017; Burgess et al., 2019). Dermal Mϕ are likely early responders to skin wounding via recognition of damage associated molecular pattern (DAMP) molecules or pathogen associated molecular pattern (PAMP) molecules (Davies et al., 2013; Malissen et al., 2014; Minutti et al., 2017). These tissue-resident Mϕ originate from yolk sac but are replenished by fetal liver-derived Mo in the embryo and by bone marrow Mo after birth. The major functions of these Mϕ are maintenance of skin homeostasis and integrity, tissue repair, and stress response (Tamoutounour et al., 2013; Ginhoux and Guilliams, 2016; Yanez et al., 2017). In addition, Langerhans cells, which are epidermal dendritic cells but share Mϕ markers such as MHC-II, F4/80 and CD14 also play important roles in wound healing (Malissen et al., 2014; Minutti et al., 2017). Langerhans cells originate both from the yolk sac during primitive hematopoiesis and fetal liver-derived Mo during definitive hematopoiesis. However, in contrast to dermal Mϕ, Langerhans cells are maintained by self-replication without any replenishment from bone marrow monocyte pool (Merad et al., 2002; Hoeffel et al., 2012, 2015; Gomez Perdiguero et al., 2015; Ginhoux and Guilliams, 2016).

Skin wounding induces a rapid, large infiltration of inflammatory Mo (CCR2^+^Ly6C^+^) into wounds followed by conversion of the Mo into Mϕ (Ly6C^–^F4/80^+^) as healing progresses (Koh and DiPietro, 2011; Willenborg et al., 2012; Crane et al., 2014; Rodero et al., 2014; Wynn and Vannella, 2016; Barman et al., 2019a, b). Blood Mo are thought to be the main source of wound Mo/Mϕ and a rapid decrease in CD11b^+^CD115^+^Ly6C^hi^ blood Mo 4–6 h post wounding correlates in time with the increase of inflammatory Mo in skin wound Mo (Rodero et al., 2014). After infiltrating wounds, novel recent findings demonstrate that inflammatory Mo/Mϕ (Ly6C^hi^F4/80^–/lo^) proliferate rapidly peaking on day 6 post-wounding. In contrast, the majority of mature wound Mϕ (Ly6C^–^F4/80^+^) remain at resting G0 phase indicating that proliferation of infiltrating inflammatory Mo followed by their differentiation into mature Mϕ results in wound Mϕ expansion (Pang et al., 2019). In addition, several studies have demonstrated that bone marrow-derived Mo contribute to skin wound Mϕ and that similar to other tissue injuries such as myocardial infarction and hindlimb ischemia, skin wounding also promotes bone marrow monopoiesis in mice (Ishida et al., 2008; Sager et al., 2015; Fang et al., 2018; Barman et al., 2019a). However, unlike myocardial infarction, skin wounding-induced monopoiesis in bone marrow occurs independently of IL-1R1 signaling (Sager et al., 2015; Barman et al., 2019a). Altogether, these data clearly suggest that there is a communication between skin wounding and bone marrow for increased Mo production which may be critical for normal wound healing.

## Mϕ Subsets in Skin Wound Healing

During the early phases of healing, wound Mo/Mϕ exhibit a pro-inflammatory or “classically activated” M1-like phenotype, which gradually transitions into a healing-associated or “alternatively activated” M2-like phenotype; it is well documented that such transition of phenotypes is essential for normal wound healing (Willenborg et al., 2012; Mirza et al., 2013, 2014; Kimball et al., 2018). In addition to the M1/M2 or related classification schemes, other phenotypically distinct Mϕ subsets have been reported to play important roles during skin wound healing. For example, dermal Mϕ identified as CD64^+^, MERTK^+^, and CCR2^–/low^ influence wound healing by their highly phagocytic nature (Malissen et al., 2014). A recent study has demonstrated two distinct Mϕ subsets in skin wounds which differ in both function and origin and are distinguishable by surface CX3CR1 staining. CX3CR1^hi^ Mϕ were derived from tissue resident Mϕ and were predominantly alternatively activated, whereas CX3CR1^–/lo^ wound Mϕ were derived from recruited Mo and exhibited both classical and alternative activation states (Burgess et al., 2019). Another subset of tissue resident Mϕ known as skin trans endothelial radio-resistant anti-inflammatory Mϕ (STREAM) were found to be located in perivascular regions and constitutively express an anti-inflammatory transcriptional profile. Interestingly, these Mϕ were resistant to polarization toward inflammatory phenotypes under inflammatory stimuli, hence appearing to be critical for tissue repair and regeneration (Barreiro et al., 2016). Similarly, another report described CD11b^+^F4/80^+^CD206^+^CD301b^+^ wound Mϕ to be critical for reparative mechanism which are increased during the proliferative phase of wound healing (Shook et al., 2016). These data suggest that there are functionally distinct Mϕ subsets in skin wounds which play critical roles at different stages of wound healing, however, how the reported Mϕ subsets may be related to each other, how each is regulated and their precise roles in healing remain to be determined.

## Mϕ Functions in Normal Wound Healing

Tissue-resident dermal Mϕ are likely among the earliest responders to skin injury, helping to induce the inflammatory response via release of hydrogen peroxide resulting in recruitment of blood neutrophils and monocytes (Davies et al., 2013; Malissen et al., 2014; Minutti et al., 2017). During early wound healing, Mo/Mϕ help to clear the wound of contaminating microbes as well as apoptotic neutrophils and cellular debris via phagocytosis (Meszaros et al., 1999, 2000; Silva, 2010; Soehnlein and Lindbom, 2010; Chen et al., 2015). The importance of Mϕ in such wound debridement is supported by studies targeting macrophage peroxisome proliferator-activated receptor γ (PPARγ), which plays a role in efferocytosis (Chen et al., 2015). PPARγ KO mice exhibit increased accumulation of apoptotic neutrophils in wounds and impaired wound healing, indicating impaired clearance of apoptotic cells. Further, treatment with a PPARγ agonist reduced accumulation of apoptotic neutrophils in wounds and improved healing (Chen et al., 2015).

Another important function of Mϕ is to regulate the activity of other wound cells via the production and release of many different cytokines and growth factors. Early after tissue injury, Mϕ release numerous inflammatory mediators including IL-1β, TNF-α, IL-6, and others to amplify the inflammatory response (Barrientos et al., 2008). In addition, wound Mo/Mϕ are an important source of growth factors such as VEGF, which is critical for angiogenesis and tissue growth (Stockmann et al., 2011; Willenborg et al., 2012). Later in the healing process, Mϕ secrete other growth factors such as TGF-β, FGF, and IGF-1 that induce cell proliferation and protein synthesis which are critical for healing (Hunt et al., 1984; Rappolee et al., 1988). Finally, Mϕ have been shown to be involved in collagen degradation during tissue remodeling phase of repair (Madsen et al., 2013; Roch et al., 2014; Wang et al., 2017). A study that depleted Mϕ in *LysM-Cre/DTR* mice at different stages of wound healing supports the notion that Mϕ change functions throughout the healing process – loss of Mϕ during early stages of healing leads to reduced epithelialization, granulation tissue formation and wound contraction whereas Mϕ depletion during the mid-phase abrogates transition of wound tissues from regeneration to maturation phase (Lucas et al., 2010). Collectively, these studies demonstrate that Mϕ play diverse roles throughout each stage of wound healing and thus are integral components of wound repair.

## Mo/Mϕ Dysregulation and Impaired Wound Healing in Diabetes

**FIGURE 1:**
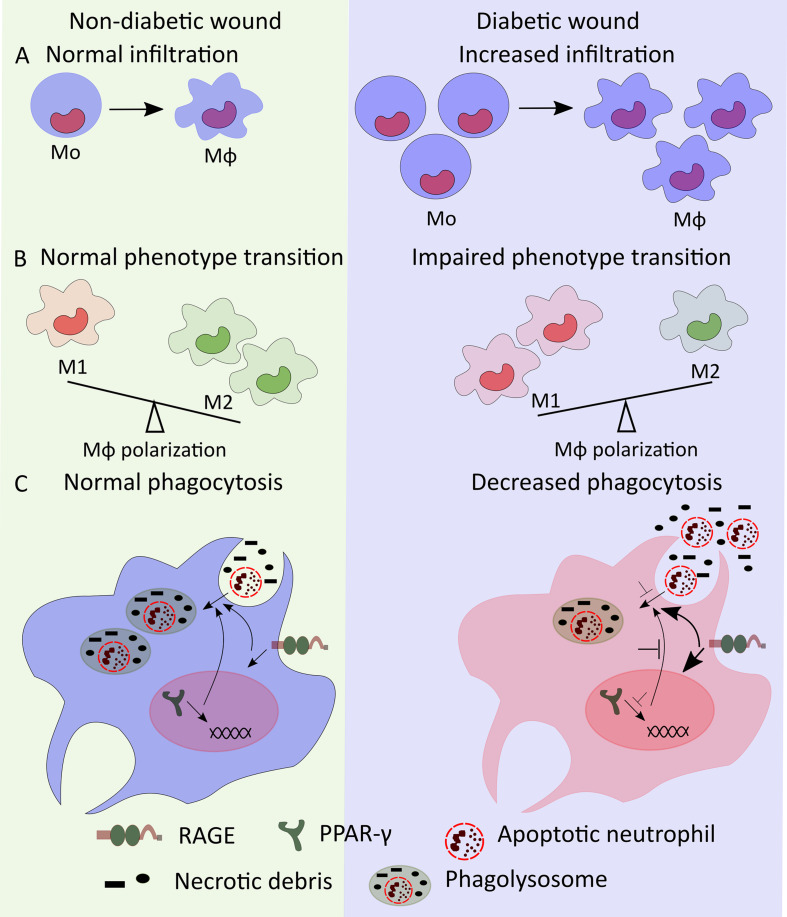
Mo/Mϕ dysregulation in diabetic wound. **(A)** High numbers of bone marrow-produced Mo may lead to increased number of diabetic wound Mϕ. **(B)** Mϕ transition from M1- to M2-like phenotypes is impaired in diabetic wounds resulting in increased accumulation of M1-like wound Mϕ. **(C)** Decreased PPAR-γ and increased RAGE signaling reduce the phagocytic ability of wound Mϕ decreasing phagocytosis of apoptotic neutrophils and necrotic debris thus leading to increased accumulation of neutrophils and necrotic debris in diabetic wounds.

Diabetes is a metabolic disorder leading to low-grade systemic inflammation which is known to have a significant impact on the immune system (Esser et al., 2014). Dysregulated metabolic pathways and host immune response contribute to impairments in each phase of wound healing, ultimately causing delayed wound healing in diabetes (Patel et al., 2019). Along with various other factors, alterations in both the number and phenotype of wound Mo/Mϕ likely contribute to impaired wound healing in diabetes. The number of infiltrating Mo is found to be higher in the wounds of leptin receptor mutant (Lepr^db^) db/db type 2 diabetic mice early after wounding (Bannon et al., 2013; Barman et al., 2019b). Following such increased monocyte accumulation, the macrophage subsets (Ly6C^+^F4/80^+^ and Ly6C^–^F4/80^+^) are also increased significantly in db/db mouse wounds at later time points suggesting a persistent Mo/Mϕ response in diabetic wounds (Mirza and Koh, 2011; Gallagher et al., 2015; Kimball et al., 2018; Barman et al., 2019b). Further, a recent study has shown that the proportion of early wound Mϕ differentiated from infiltrating bone marrow-derived monocytes in db/db diabetic mice are increased whereas wound Mϕ derived from tissue resident Mϕ remain unaltered as compared to non-diabetic wounds (Burgess et al., 2019). Impaired wound healing in high-fat-diet induced obese (DIO) pre-diabetic mice is also associated with persistent accumulation of inflammatory Mo/Mϕ in non-healing wounds (Gallagher et al., 2015; Kimball et al., 2018). Prolonged infiltration of blood Ly6C^hi^ Mo is thought to be responsible for the sustained accumulation of inflammatory Mo/Mϕ in the skin wounds of DIO mice. Such extended infiltration of inflammatory Mo may contribute to the observed defect in the transition from Ly6C^hi^ into Ly6C^lo^ Mo/Mϕ phenotypes (Kimball et al., 2018) (Figure 1A). In contrast to these reports, there are also reports of decreased numbers of wound Mo/Mϕ early after wounding in diabetic mice attributed in part to an early impairment of chemotaxis into the wound (Wood et al., 2014; Yan et al., 2018). The discrepancy in wound macrophage numbers between studies could result from technical differences in the assessment of wound Mϕ and deserves further study.

Wound Mo/Mϕ in diabetic mice persistently express high levels of M1-like Mϕ markers such as NOS2, TNF-α, IL-1β, MMP9, and low levels of M2-like Mϕ markers such as Arginase 1, CD206, CD36 (Mirza and Koh, 2011; Bannon et al., 2013; Mirza et al., 2013, 2014). Diabetic wound-derived Mϕ also exhibit decreased expression of pro-healing factors such as IGF-1, TGF-β and VEGF (Mirza and Koh, 2011; Mirza et al., 2013, 2014). Similar to diabetic mice, wound biopsies from human diabetic foot ulcers also show increased proportion of M1-like Mϕ (CD68^+^ and IL-1β^+^) and decreased proportion of M2-like Mϕ (CD163^+^, CD206^+^, and Arginase-1^+^), respectively (Figure 1B) (Bannon et al., 2013; Mirza et al., 2013; Gallagher et al., 2015). The sustained pro-inflammatory phenotype of diabetic wound Mϕ likely helps to drive a persistent pro-inflammatory microenvironment in diabetic wounds characterized by increased levels of IL-1β, IFN-γ, TNF-α, and IL-12 as well as decreased levels of pro-healing factors such as IGF-1, TGF-β1, VEGF, and IL-10 (Mirza and Koh, 2011; Bannon et al., 2013; Mirza et al., 2013). Together, these reports indicate that dysregulation of Mϕ polarization likely plays critical roles in impaired diabetic wound healing in both mice and humans.

There is also consensus in the literature that wound Mo/Mϕ in diabetic mice and humans show impaired phagocytosis that may contribute to impaired wound healing (Khanna et al., 2010; Pavlou et al., 2018). Wound Mϕ in diabetic mice exhibit reduced phagocytosis resulting in increased accumulation of apoptotic cells in the wounds and a sustained pro-inflammatory microenvironment (Khanna et al., 2010). In addition, reduced PPAR-γ expression in diabetic wound Mϕ and improved wound healing in diabetic mice with topical wound treatment with PPAR-γ agonist is consistent with the idea that PPAR-γ-mediated macrophage clearance of apoptotic wound neutrophils may play an important role in wound healing (Khanna et al., 2010; Chen et al., 2015; Mirza et al., 2015). In another report, antibody mediated topical inhibition of receptor for advanced glycation end products (RAGE) signaling showed reduced number of neutrophils in diabetic wounds in association with enhanced phagocytosis by Mϕ and improved wound healing further supporting the notion that increased neutrophil accumulation in diabetic wounds results from reduced phagocytic ability of Mϕ which is closely associated with impaired wound healing in diabetes (Figure 1C) (Wang et al., 2017). Together, these reports suggest that decreased efferocytosis by wound Mϕ is critical for impaired wound healing in diabetes.

Dysregulated Mϕ activity in diabetic wounds impairs processes critical for normal wound healing. For example, Mϕ are known to play important roles in neovascularization during wound healing, and decreased production of VEGF-A as well as reduced VEGFR1 signaling by diabetic wound Mϕ likely contribute to impaired angiogenesis in diabetic wounds (Stockmann et al., 2011; Willenborg et al., 2012; Okizaki et al., 2016; Okonkwo and DiPietro, 2017; Gurevich et al., 2018; Okonkwo et al., 2020). Mϕ likely also play important roles in the impaired maturation and remodeling of the vasculature in diabetic wounds. Altogether, the available data suggest that dysregulation of wound Mo/Mϕ both in terms of numbers and phenotypes plays critical roles in impaired diabetic wound healing.

## Diabetes-Induced Alteration of Mo Production

**FIGURE 2:**
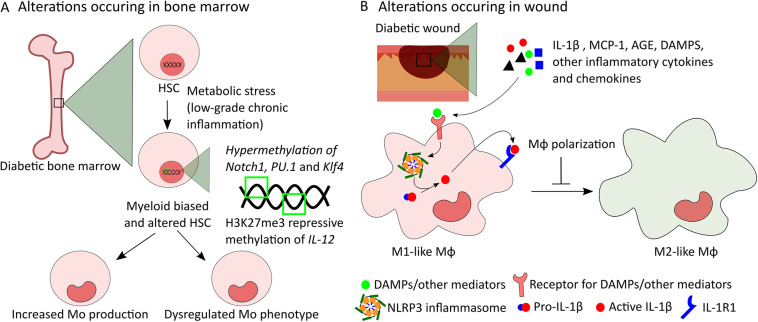
Potential mechanisms of alterations in bone marrow-derived Mo and in wound Mϕ in diabetes. **(A)** Low-grade chronic inflammation caused by metabolic stress induces myeloid bias in diabetic HSCs leading to increased production of Mo. In addition, intrinsic modifications of HSCs caused by epigenetic changes such as hypermethylation of *Notch1*, *PU.1*, and *Klf4* genes and H3K27me3 repressive methylation of *IL-12* gene can be passed down to Mo altering their phenotype. **(B)** Increased levels of IL-1β, MCP-1, AGE, DAMPs, and other inflammatory cytokines and chemokines in wound microenvironment induce NLRP3 and IL-1R1 signaling and other inflammatory pathways in wound Mϕ leading to dysregulated polarization of Mϕ from M1 to M2-like phenotype in diabetic wounds.

Several reports have shown that diabetes increases monopoiesis in the bone marrow of STZ-induced and Akita (*Ins2*^Akita^) type 1 diabetic mice where pancreatic β cells are destroyed by toxic effects of STZ or misfolded insulin (Nagareddy et al., 2013), DIO pre-diabetic mice (Singer et al., 2014), and db/db type 2 diabetic mice at steady state (Nagareddy et al., 2014; Barman et al., 2019b). STZ-induced diabetes and DIO mice also exhibit extramedullary myelopoiesis in the spleen resulting in increased number of splenic Mo during homeostasis (Vasamsetti et al., 2018). Furthermore, diabetic patients have been shown to display elevated number of circulating inflammatory Mo (CD14^+^CD16^–^) indicating diabetes-induced enhanced monopoiesis in human (Vasamsetti et al., 2018). However, skin wounding-induced Mo expansion in mouse bone marrow is not further augmented by diabetes (Barman et al., 2019b). Collectively, these data suggest increased steady-state monopoiesis in diabetes likely contributes to enhanced wound Mo/Mϕ and impaired wound healing (Figure 2A).

Increased monopoiesis in diabetes is associated with modification of the hematopoietic stem and progenitor cell (HSPC) compartment (Ferraro et al., 2011; Gallagher et al., 2015; Lee et al., 2018; Vasamsetti et al., 2018; Barman et al., 2019b). Recently, bone marrow-derived myeloid progenitors (LK, Lin^–^cKit^+^Sca-1^–^ cells) from db/db mice have been shown to be intrinsically modified to produce increased number of Mo upon IL-1β or M-CSF challenge *in vitro* corroborating enhanced potential of diabetic myeloid progenitors to Mo production (Barman et al., 2019b). Alterations of the HSPC niche, which regulates HSPC maintenance, mobilization and differentiation, likely play an important role in the alterations of HSPC phenotypes in diabetes (Ferraro et al., 2011; Lucas et al., 2013; Morrison and Scadden, 2014; Boulais and Frenette, 2015; Vasamsetti et al., 2018; Albiero et al., 2019). Increased level of sympathetic nerves in the HSC niche is believed to induce the alterations in niche components in diabetes (Ferraro et al., 2011; Vasamsetti et al., 2018).

Importantly, increased IL-1R1 signaling in myeloid restricted bone marrow progenitors may be responsible for increased monopoiesis in obese and pre-diabetic mice (Nagareddy et al., 2014). In these studies, IL-1β produced by adipose Mϕ is thought to mediate communication between adipose tissue and bone marrow. However, diabetic db/db recipient mice transplanted with *Il1r1^–/–^* or WT donor cells showed no difference in myeloid cell output indicating that IL-1R1 signaling is likely not involved in diabetes-associated increased myelopoiesis in the db/db mouse model (Barman et al., 2019b). These seemingly disparate roles of IL-1R1 signaling in increased myelopoiesis in different animal models of pre-diabetes and diabetes deserve further study.

In addition, RAGE has also been shown to be associated with increased monopoiesis in other models of diabetes such as STZ-induced and Akita diabetic mice (Nagareddy et al., 2013). Neutrophil-derived S100A8/A9 mediated activation of NF-kB-dependent RAGE signaling in common myeloid progenitors (CMPs) which in turn induces GMP proliferation via growth factors leads to increased monopoiesis in these diabetic mice (Nagareddy et al., 2013). Taken together, these data suggest that IL1-R1 and RAGE signaling may act as intrinsic drivers to promote monopoiesis in pre-diabetic and diabetic mice.

## Wound Microenvironment and Bone Marrow Progenitor Modifications Influence Diabetic Wound Mϕ

Mϕ phenotypes in diabetic wounds can be altered both through local effects mediated by the wound microenvironment and through epigenetic modifications that may occur in bone marrow progenitors that are passed down to macrophage progeny (Mirza et al., 2013, 2014; Gallagher et al., 2015; Yan et al., 2018). There is strong evidence in the literature supporting the importance of the local microenvironment in determining macrophage phenotypes. First, the ability of diabetic wound conditioned medium to induce M1-like phenotypes in bone marrow-derived Mϕ *in vitro* supports the hypothesis that diabetic wound microenvironment may play important roles in determining Mϕ phenotypes (Mirza et al., 2013, 2014). Studies showing that local modification of diabetic wound microenvironment by blocking IL-1β or RAGE, or inhibiting inflammasome pharmacologically can shift wound Mϕ toward pro-healing phenotypes further support the notion that wound microenvironment has significant effect on dysregulation of Mϕ phenotypes in diabetic wounds and that sustained activation of NLRP3 (NLR family, pyrin domain-containing 3) inflammasome or of the RAGE pathway in wound Mo/Mϕ is likely involved (Figure 2B) (Mirza et al., 2013, 2014; Wang et al., 2017). Further, the level of MCP-1 is found to be higher in the wounds of DIO mice and antibody mediated inhibition of this chemokine during the inflammatory phase of wound healing normalizes the number of inflammatory wound Mo/Mϕ and improves wound healing (Kimball et al., 2018). Together, these data suggest that wound microenvironment may play important roles in dysregulated Mϕ functions in diabetic wounds.

Intrinsic modifications of bone marrow progenitors that are passed down to Mo/Mϕ may also contribute to dysregulated Mϕ phenotypes in diabetic wounds (Bannon et al., 2013; Gallagher et al., 2015; Yan et al., 2018; Davis and Gallagher, 2019). For example, differential responses to classical and alternate activation *in vitro*, amplified pro-inflammatory phenotypes and sustained potential to produce inflammatory tissue Mϕ observed in bone marrow-derived cells from different diabetic mouse models suggest that diabetes functionally alters progenitors in the bone marrow which may lead to dysregulated Mϕ phenotypes in diabetic wounds (Nagareddy et al., 2014; Singer et al., 2014). Additional studies indicated that epigenetic modifications of HSPCs may be involved in such phenotypic alterations in diabetic bone marrow progenitors (Gallagher et al., 2015; Yan et al., 2018; Davis and Gallagher, 2019). For example, epigenetic modification of HSPCs by means of decreased repressive histone methylation mark H3K27me3 at the IL-12 gene promoter has been shown to be passed down to wound Mϕ in DIO mice resulting in increased pro-inflammatory wound Mϕ and impaired wound healing (Gallagher et al., 2015). In addition, transplantation of diabetic hematopoietic stem cells (HSCs) from db/db into WT mice has been shown to cause delayed wound healing in association with sustained accumulation of M1-like wound Mϕ indicating that dysregulation of diabetic wound Mϕ phenotypes happens via a HSC autonomous mechanism (Yan et al., 2018). Epigenetic modification of myeloid lineage associated genes such as *Notch1*, *PU.1* and *Klf4* by DNA methyltransferase 1 (Dnmt1) mediated hypermethylation may induce such alterations in diabetic HSCs (Figure 2A) (Yan et al., 2018). Altogether, these data suggest both wound microenvironment-mediated alteration of wound Mϕ and epigenetic modification of HSPCs as potential mechanisms of dysregulated Mϕ phenotypes in diabetic wounds.

## Conclusion, Implications, and Future Directions

In summary, both sustained increases in the number of wound Mo/Mϕ and dysregulation of their phenotype, caused both by intrinsic alterations in bone marrow progenitors and by a pro-inflammatory wound microenvironment, lead to impaired wound healing in diabetes (Mirza et al., 2013, 2014; Nagareddy et al., 2014; Singer et al., 2014; Gallagher et al., 2015; Yan et al., 2018). Improved understanding of factors that regulate wound Mo/Mϕ numbers and phenotype have led to new therapeutic interventions attempting to normalize the Mϕ response in mice targeting the NLRP3 inflammasome/IL-1β and RAGE pathways; these findings await translation to humans (Mirza et al., 2013; Wang et al., 2017). Another approach for normalizing Mϕ phenotypes in non-healing wounds could be altering epigenetic modifications of genes associated with dysregulated Mϕ phenotype (Gallagher et al., 2015; Yan et al., 2018). However, more comprehensive knowledge on epigenetic changes that drive persistent inflammation in diabetic wound Mo/Mϕ will be useful to specifically target relevant genes.

Studies showing that diabetes-associated increased monopoiesis at steady-state contributes to increased Mϕ accumulation in diabetic wounds suggest the possibility that targeting monopoiesis may help normalizing wound Mϕ accumulation and improve diabetic wound healing (Barman et al., 2019b). However, much remains to be learned about the regulation of monopoiesis during wound healing.

Lastly, several studies have highlighted the importance of Mo subsets in health and disease (Wolf et al., 2019). Heterogenous Mo/Mϕ populations have also been identified in various tissues such as lung (Mould et al., 2019), aorta (Cochain et al., 2018), and heart (Dick et al., 2019) under diseased conditions. However, our knowledge of the heterogeneity of skin wound Mϕ is lacking, especially during impaired healing. Multiplex analyses such as single cell RNA-sequencing, multiparameter flowcytometry, and imaging mass cytometry will be helpful to acquire knowledge on Mϕ subsets present during wound healing, and how these are regulated during normal and impaired wound healing which may, in turn, provide insight into new approaches for manipulating inflammation and improving healing.

## Author Contributions

PB and TK have made a substantial, direct and intellectual contribution to the work. Both authors contributed to the article and approved the submitted version.

## Conflict of Interest

The authors declare that the research was conducted in the absence of any commercial or financial relationships that could be construed as a potential conflict of interest.

## Abbreviations

CMP: common myeloid progenito
GMP: granulocyte macrophage progenitor
DIO: diet-induced obese
HSC: hematopoietic stem cells
HSPC: hematopoietic stem and progenitor cell
IL-1R1: interleukin-1 receptor 1
Mo/M ϕ: monocyte/macrophage
PPAR γ: peroxisome proliferator-activated receptor γ
RAGE: receptor for advanced glycation end products
STZ: streptozotocin.
